# Primary myeloma interaction and growth in coculture with healthy donor hematopoietic bone marrow

**DOI:** 10.1186/s12885-015-1892-7

**Published:** 2015-11-06

**Authors:** Rakesh Bam, Sharmin Khan, Wen Ling, Shelton S. Randal, Xin Li, Bart Barlogie, Ricky Edmondson, Shmuel Yaccoby

**Affiliations:** Myeloma Institute for Research and Therapy, University of Arkansas for Medical Sciences, Little Rock, AR USA

**Keywords:** Myeloma, Microenvironment, Bone marrow

## Abstract

**Background:**

Human primary myeloma (MM) cells do not survive in culture; current in vitro and in vivo systems for growing these cells are limited to coculture with a specific bone marrow (BM) cell type or growth in an immunodeficient animal model. The purpose of the study is to establish an interactive healthy donor whole BM based culture system capable of maintaining prolonged survival of primary MM cells. This normal BM (NBM) coculture system is different from using autologous BM that is already affected by the disease.

**Methods:**

Whole BM from healthy donors was cultured in medium supplemented with BM serum from MM patients for 7 days, followed by 7 days of coculture with CD138-selected primary MM cells or MM cell lines. MM cells in the coculture were quantified using flow cytometry or bioluminescence of luciferase-expressing MM cells. T-cell cytokine array and proteomics were performed to identify secreted factors.

**Results:**

NBM is composed of adherent and nonadherent compartments containing typical hematopoietic and mesenchymal cells. MM cells, or a subset of MM cells, from all examined cases survived and grew in this system, regardless of the MM cells’ molecular risk or subtype, and growth was comparable to coculture with individual stromal cell types. Adherent and nonadherent compartments supported MM growth, and this support required patient serum for optimal growth. Increased levels of MM growth factors IL-6 and IL-10 along with MM clinical markers B2M and LDHA were detected in supernatants from the NBM coculture than from the BM cultured alone. Levels of extracellular matrix factors (e.g., MMP1, HMCN1, COL3A1, ACAN) and immunomodulatory factors (e.g., IFI16, LILRB4, PTPN6, AZGP1) were changed in the coculture system. The NBM system protected MM cells from dexamethasone but not bortezomib, and effects of lenalidomide varied.

**Conclusions:**

The NBM system demonstrates the ability of primary MM plasma cells to interact with and to survive in coculture with healthy adult BM. This model is suitable for studying MM-microenvironment interactions, particularly at the early stage of engagement in new BM niches, and for characterizing MM cell subpopulations capable of long-term survival through secretion of extracellular matrix and immune-related factors.

**Electronic supplementary material:**

The online version of this article (doi:10.1186/s12885-015-1892-7) contains supplementary material, which is available to authorized users.

## Background

Multiple myeloma (MM) is a genetically heterogeneous hematological cancer in which malignant plasma cells proliferate in and destroy the bone marrow (BM). Understanding the complex interactions between these MM cells and the cells in their microenvironment is crucial for developing new therapies targeting MM and its associated bone disease.

Although in vitro and in vivo studies of MM cell lines are abundant, studies of primary MM cells are limited, often due to difficulties with maintaining primary MM outside the functional BM. Survival and growth of primary human MM cells and cell lines have been demonstrated in coculture with mesenchymal cells [1–5], osteoblasts [6], osteoclasts [7], macrophages [8], and dendritic cells [9]. A 3D autologous BM system, containing microenvironmental cells already affected by the disease, has been established to study survival of MM cells and their precursors in vitro [10]. Growth of human primary MM plasma cells has also been demonstrated in animal models, including SCID-hu [11, 12], SCID-rab [13–15], and scaffold-based [16] models, and growth of so-called MM stem cells—either clonotypic B lymphocytes or phenotypically immature MM cells—has been demonstrated in nonobese diabetic/severe combined immunodeficiency (NOD/SCID) mice [17, 18]. Almost all in vivo models that use human MM cells have performed studies in immunodeficient mice that fail to exhibit immune cell-driven tumor growth dynamics. In contrast, certain in vivo murine MM models such as the 5TMM model [19] or transgenic mouse models [20] have been successfully exploited for studying MM-immune cell interaction and immunotherapy [21, 22].

To fill the existing need, we developed a novel comprehensive in vitro coculture model—the normal bone marrow (NBM) system—that contains cell types of healthy donor BM, including immune cells, which are precultured with serum from MM patients before being used for coculture with primary MM plasma cells. This NBM model is different from previous models that coculture MM cells with single types of BM cells [7] or that use autologous BM already affected by the disease without consideration of the heterogeneous nature of MM [10]. We demonstrate here that the NBM system consistently supports long-term survival of primary MM plasma cells. Our work establishes an in vitro culture system to mainly maintain primary MM cells and identifies some factors pertinent to MM survival in a whole-BM microenvironment.

## Methods

### Primary MM cells and MM cell lines

Primary MM cells were obtained from heparinized BM aspirates from patients with active MM during scheduled clinic visits. Signed Institutional Review Board–approved informed consent forms are on record. BM samples were separated by density centrifugation with Ficoll-Paque (specific gravity, 1.077 g/ml; Amersham Biosciences, Piscataway, NJ). Plasma cells were isolated with CD138 immunomagnetic bead selection and autoMACs automated separation system (Miltenyi-Biotec, Auburn, CA). Purity of CD138^+^ primary samples was routinely >90 %, as assessed by flow cytometry [7].

For in vitro bioluminescence-based assays of patients’ MM cells, we used patients’ cases in which their MM cells were capable passaging in SCID-rab mice as described [23]. Passaged MM cells were plated in 24-well plate (100,000 cells/well) and transduced by adding the lentiviral particles expressing both enhanced green fluorescent protein (EGFP) and luciferase to cell suspensions at MOI (multiplicity of infection) of 2 for 24 h [23], followed by engraftment of these cells in SCID-rab mice. Following establishment of high tumor burden as assessed by periodic measurement of human immunoglobulins in mice sera and by live-animal imaging [23], tumor cells were then extracted from the implanted bone and EGFP/luciferase-expressing MM cells were sorted by FACS based on EGFP expression. The sorted MM cells were subsequently engrafted and passaged in SCID-rab mice. EGFP/luciferase-expressing MM cells extracted from these mice were used in indicated coculture experiments.

The stroma-dependent JB MM cell line was established at our institute and grown as described [23]. The stroma-independent cell line OPM2 was obtained from American Type Culture Collection (ATCC; Manassas, VA) and ARP1 line was established in our institute.

### NBM coculture system

**Fig. 1:**
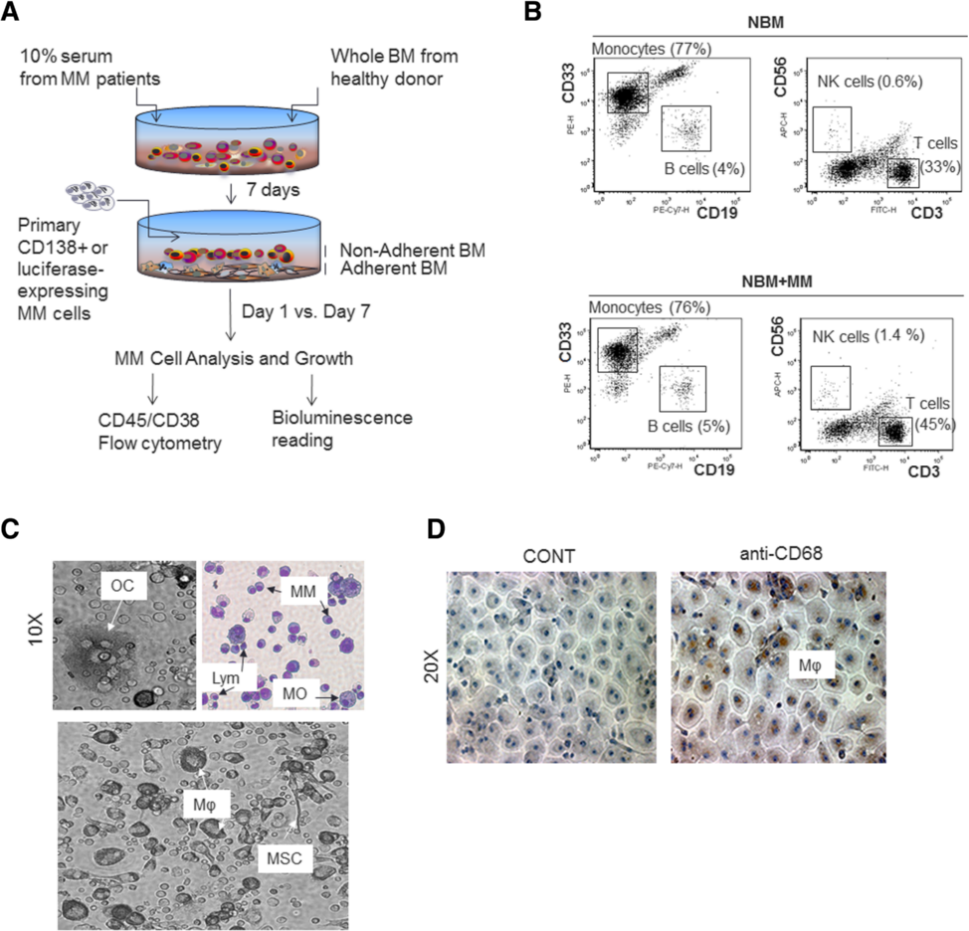
Characterization of the NBM coculture system. **a** A schematic representation of NBM coculture system, showing formation of adherent and nonadherent BM compartments upon culture with patient serum. In this system, MM cell growth is measured with CD45/CD38 flow cytometry or direct bioluminescence of luciferase-expressing MM cells from day 1 to 7 of coculture. At these time points, conditioned medium and distinct cellular compartments including MM cells can be collected or purified for further analysis. **b** Prior to initiation of coculture, whole BM cells from healthy donors were cultured for 7 days in medium supplemented with MM patients’ serum; this was followed by 7 days without (*top panel*, NBM) or with (*bottom panel*, NBM + MM) MM cells. The whole BM was subjected to flow cytometry analysis of CD3 T cells, CD19 B cells, CD33 monocytes and CD56 NK cells. Percent values of each type of the cell in the NBM culture are shown. **c** Phase contrast images from coculture system (10X magnification): (*upper left panel*) multinucleated osteoclast-like cell (OC); (*lower panel*) cells that resemble macrophages (Mφ) and mesenchymal stem cells (MSC). Giemsa staining of nonadherent cells (*upper right panel*) from coculture system: typical hematopoietic cells such as lymphocytes (Lym) and monocytes (MO), and MM plasma cells (MM). **d** Phase contrast images (20X magnification) of adherent BM cells from coculture system: immunocytochemical staining of macrophages (Mφ) expressing CD68 (*right*)

Preparation of the NBM system is illustrated in Fig. 1a. Freshly obtained (via overnight shipping) whole BM cells from healthy adult (>40 years old) donors (*n* = 20, Allcells, Alameda, CA) were lysed with RBC lysis buffer (Qiagen Sciences, Germantown, MD) for 20 min on ice. Cells were centrifuged, and pellets were washed three times in phosphate-buffered saline (PBS) and resuspended (2 × 10^6^ cells/ml) in αMEM medium (Gibco, Grand Island, NY) containing 10 % fetal bovine serum and 10 % serum from MM patients. Patient serum was used to pre-condition the NBM culture and emulate a supportive microenvironment before adding MM cells. Typically, serum from patients with active MM (mostly newly diagnosed cases) was pooled and aliquots were stored at −80 °C for availability. BM cells were seeded on a 24-well plate (2 × 10^6^ cells/well in 1 ml) and 96-well white plate (400,000 cells/well in 200 μl) and were incubated for 7 days at 37 °C; MM plasma cells then were added to each well (4 BM cells: 1 MM cell). In general, each donor’s BM sample was used for running >15 coculture experiments. MM cells were quantified with flow cytometry on days 1 and 7 or with bioluminescence (Veritas microplate luminometer; Turner Biosystems, Sunnyvale, CA) on days 1 and 5. Testing anti-MM clinical agents in the NBM system was based on the bioluminescence method using EGFP/luciferase expressing MM cells.

### Flow cytometry

BD FACSVerse (BD Biosciences, San Diego, CA) was used for flow cytometric analyses. For phenotypic examination of normal BM culture, nonadherent cells were collected and remaining adherent cells were incubated in 0.5 ml StemPro Accutase cell dissociation reagent (Life Technologies, Grand Island, NY) for 10–20 min at 37 °C. These detached adherent cells were then added to previously collected nonadherent cells. Cells were washed with PBS, resuspended in incubation buffer (0.5 % bovine serum albumin [BSA] in PBS), and stained with multicolor markers (FITC-anti-CD3, PE-anti-CD33, PE-Cy7-anti-CD19, APC-anti-CD56; BD Biosciences) for 15 min at room temperature in the dark. Cells were washed and resuspended in 400 μl PBS before analysis.

To identify MM plasma cells in coculture with normal BM, total cells were collected as described above. Cell aliquot was stained with 0.02 % crystal violet (LabChem Inc, Pittsburg, CA) for 10 min and counted on a hemocytometer. MM cells were delineated from BM cells with staining for PE-anti-CD38 and FITC-anti-CD45 (BD Biosciences) as described [7, 24]. Percent plasma cells based on CD45/CD38 staining, was used to calculate the absolute number of MM cells from the counted total number of cells of the corresponding sample.

Cell membrane labeling dye PKH26 (Sigma Aldrich, St. Louis, MO) was used to show sequential loss of signal in proliferating MM cells in the NBM coculture system.

### Immunocytochemistry

NBM cells were cultured for 7 days on chamber slides (Lab-tek, Rochester, NY). Nonadherent cells were washed away, and adherent cells were fixed with HistoChoice (AMRESCO, Solon, OH), followed by antigen retrieval using a water bath at 80 °C for 25 min. After peroxidase quenching with hydrogen peroxide (3 %) for 5 min, fixed cells were incubated with mouse anti-human CD68 (Abcam, Cambridge, MA) for 60 min, and the assay was completed with Dako’s immunoperoxidase kit (Dako Corp., Carpintera, CA). Cells were lightly counterstained with hematoxylin. An Olympus BH2 microscope (Melville, NY) was used to obtain images with a SPOT 2 digital camera (Diagnostic Instruments, Inc., Sterling Heights, MI). Adobe Photoshop version 10 (Adobe Systems, San Jose, CA) was used to process the images.

### 5-bromo-2-deoxyuridine (BrdU) labeling index

APC BrdU Flow Kit (BD Biosciences) was used to compare cell proliferation. At the end of 7 days, cocultured cells were incubated with 10-μM BrdU for 2 h. MM cells were separated from the NBM coculture by using CD138-immunomagnetic beads and then fixed, permeabalized, treated with DNase (to expose incorporated BrdU), and stained with fluorochrome-conjugated anti-BrdU antibody. MM cells separated from coculture but not exposed to BrdU were used as a control. BrdU labeling was measured with flow cytometry.

### Osteoclasts and mesenchymal stem cell (MSC) cultures

Cultures of osteoclasts and MSCs were prepared as previously described [1, 7]. For comparing growth of MM cells in different coculture systems, indicated luciferase-expressing MM cells (10^4^ cells/well) were cultured alone or cocultured for 4 days with NBM cells, osteoclasts or MSCs in 96-well plates in αMEM medium supplemented with 10 % serum pooled from MM patients (10 wells/group/cell line). Growth of MM cells was assessed with bioluminescence assays, as described above.

### NBM coculture system cytokine secretion

Conditioned medium was collected after 7 days of culturing NBM with MM patients’ serum in the absence (BM alone) and presence (NBM coculture) of CD138^+^ MM cells. Multi-Analyte ELISArray Kit (Qiagen, Valencia, CA) was used to analyze IL-2, IL-4, IL-5, IL-6, IL-10, IL-12, IL-13, IL-17A, IFN-γ, TNF-α, G-CSF and TGF-β1 levels in the culture supernatant. Briefly, supernatant samples (50 μl) were incubated for 2 h in microplates precoated with capture antibodies, followed by steps provided in the manufacturer’s protocol. Plate absorbance was measured at 450 nm, and background was subtracted with readings at 570 nm.

### Identification of secreted factors in the NBM system by proteomics

MM cells were cultured alone or with the established NBM cells (NBM/MM coculture). Separate wells of BM cells cultured alone (without MM cells) served as a control. All three groups contained patient serum from the same source. The experiment was repeated three times using three different patient MM cells and BM cells from three healthy donors. In each set of experiments, after 3 days of coculture, nonadherent cells were collected, washed three times with PBS, and added back to the original wells containing adherent cells (which were also washed three times with PBS) in phenol red free αMEM medium (Gibco) in serum-starved condition. Similar wash was performed for MM cells cultured alone or BM cells cultured without MM cells. After 18 h of culture, the supernatant from each group (MM cells, BM cells or NBM/MM coculture) was collected and subjected to proteomic analysis. After concentrating proteins from equal volumes of the supernatant samples from each group, samples were electorphoresed on a gel, lanes were cut from the gels, gel pieces were trypsin digested, and resulting samples were loaded on a mass spectrometer. The final analysis considered proteins that were significantly different (*p* < 0.05 by *t*-test) in the supernatant samples of NBM/MM coculture than in MM cells or BM cells cultured alone.

### Statistical analyses

All values are expressed as mean ± standard error of the mean, unless indicated otherwise. Student’s *t*-test or unpaired *t*-test were used to analyze the effect of anti-MM agents in the NBM system.

## Results

### Characterization of MM cell coculture with BM established from healthy donor

**Fig. 2:**
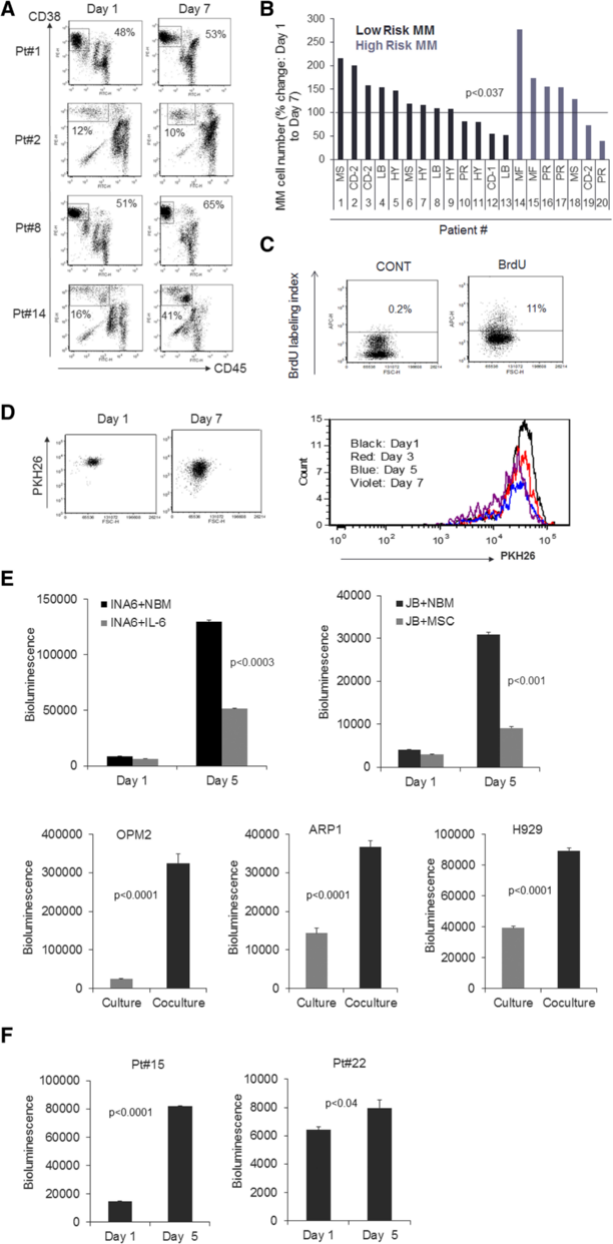
Patients’ MM cells survive and grow in NBM coculture system, regardless of relevant molecular features. **a** Four representative patient samples analyzed by CD45/CD38 flow cytometry to determine percent MM cells in the NBM coculture system on days 1 and 7. **b** Changes in number of MM cells after 7 days compared to day 1 of coculture. MM cells from 20 patients characterized according to molecularly defined risk (low or high) [26] and molecular subtype (MS, CD-1, CD-2, LB, HY, PR, MF) [25]. In 14 of 20 experiments, the number of MM cells was 59 ± 12 % higher on day 7 than on day 1 (*p* < 0.0005). *p* < 0.037 for all 20 samples. **c** Incorporation of BrdU (detected by flow cytometry) in MM cells separated from the coculture system after 7 days. Coculture was unexposed (CONT) or exposed to BrdU for 2 h before analysis. **d** (*Left panel*) Flow cytometry analysis of membrane-bound PKH26 dye in CD45/CD38 gated MM cells (Pt#18) in the NBM coculture system on days 1 and 7, showing the loss of PKH26 signal in the proliferating MM cells. (*Right panel*) Histogram of PKH26 dye from CD45/CD38 gated MM cells on day 1, 3, 5 and 7 of coculture; decreasing heights of histogram peaks indicate that most MM cells disappear over time, but a small subpopulation persists and proliferates. **e** Bioluminescence analysis of luciferase-expressing stroma-dependent MM cell lines (INA6, JB) cocultured in the NBM system for 1 and 5 days and of luciferase-expressing standard MM lines (OPM2, ARP1, H929) cocultured in the NBM system (*black* bars) or cultured in their standard conditions (*gray* bars) for 5 days. **f** Bioluminescence analysis of luciferase-expressing patients’ MM cells on days 1 and 5 in the NBM coculture system

The NBM coculture system was established for studying survival and growth of primary MM cells within a hematopoietic BM and is composed of adherent and nonadherent healthy adult BM cells and serum pooled from MM patients (Fig. 1a). Flow cytometry revealed that the nonadherent cell compartment contained typical hematopoietic cells, such as CD33^high^ monocytes, CD19^+^ B lymphocytes, CD3^+^ T lymphocytes and CD56^+^ natural killer cells (Fig. 1b). The adherent cell compartment mainly contained cells morphologically resembling macrophages, as well as few osteoclasts, megakaryocytes and MSCs (Fig. 1c). Immunohistochemically staining for CD68 confirmed abundancy of macrophages in the adherent BM compartment (Fig. 1d). At this point, after the healthy donor BM cells had been cultured for 7 days with patient serum, freshly obtained CD138-selected primary MM cells [25] were added to the cultures (ratio of 4 BM cells: 1 MM cell). After 7 days of coculture, survival of MM cells was determined by assessing the proportion of MM plasma cells to the total number of cells in the culture. MM plasma cells were identified by low to intermediate expression of CD45 together with high expression of CD38. Lack of expression of CD19 in MM cells and their position remained similar after coculture. We examined MM cells from 60 patients; a significant proportion of MM cells survived in all cases, regardless of the molecular features of the patient’s MM cells [25, 26] (Fig. 2a and b), and viability of MM cells and BM cells during the span of these experiments was high (>90 %) as assessed by trypan blue exclusion (data not shown). The NBM cultures were prepared from more than 20 healthy donors, and MM cells from several patients were typically cocultured and tested with NBM cultures from each donor. Using CD138 as another marker for quantifying MM cells produced results consistent with those based on CD45/CD38 analysis (Additional file 1: Figure S1).

### Survival and growth of primary MM cells and cell lines were variable in the NBM system

To assess survival and growth of primary MM cells, we compared the total number of MM cells on days 1 and 7 (Patients 1–20, Additional file 2: Table S1). Overall, the numbers of MM cells were significantly different between day 1 and 7 (*p* < 0.037). Moreover, in 14 of 20 experiments, the number of MM cells was 59 % (±12 %) higher on day 7 (*p* < 0.0005, *n* = 14) (Fig. 2b), reflecting the heterogeneous nature of the disease as seen in patients’ characteristics (Additional file 2: Table S1). MM cell proliferation on day 7 was evident by BrdU incorporation in MM cells separated from the coculture system (Fig. 2c) and by the loss of cell membrane dye PKH26 in cocultured MM cells (Fig. 2d). In the NBM coculture system, primary MM cells were also capable of prolonged survival (28 days) through multiple passages in freshly established NBM cultures (Additional file 3: Figure S2A). These data validate the NBM system, indicating that primary MM cells are capable of survival and growth in coculture with adult human BM containing various types of immune cells.

Because MM cells are known to closely interact with cellular elements within the BM microenvironment [7], use of the NBM system was further analyzed with bioluminescent MM cell lines, which is an efficient approach to examine tumor growth and effects of anti-MM drugs [2, 27]. Our bioluminescent MM cell lines, constructed with lentiviral particles containing an EGFP/luciferase construct, allow tumor cell growth to be monitored without separating the cells. Bioluminescent MM cells that were used included IL-6-dependent INA6 cell line; stroma-dependent JB cell line [23]; stroma-independent OPM2, ARP1 and H929 cell lines; and two patients’ MM cells that are passaged in SCID-rab mice [23]. INA6, JB, OPM2, ARP1 and H929 cell lines had higher growth rates in the NBM coculture system than under their standard growth conditions (Fig. 2e). Bioluminescence of the patients’ MM cells was also higher on day 5 than on day 1 (Fig. 2f) indicating MM growth.

To determine whether MM cell growth was affected by the source of serum (i.e., patients’ versus healthy donors’) used in the NBM system, serum from healthy donors was substituted into the model (all other parameters were unchanged). On day 7, cocultures that used serum from healthy donors had fewer MM cells than did those that used serum from MM patients (Additional file 3: Figure S2B). This indicates that patient serum is an important component in this coculture model for maintaining MM cells.

**Fig. 3:**
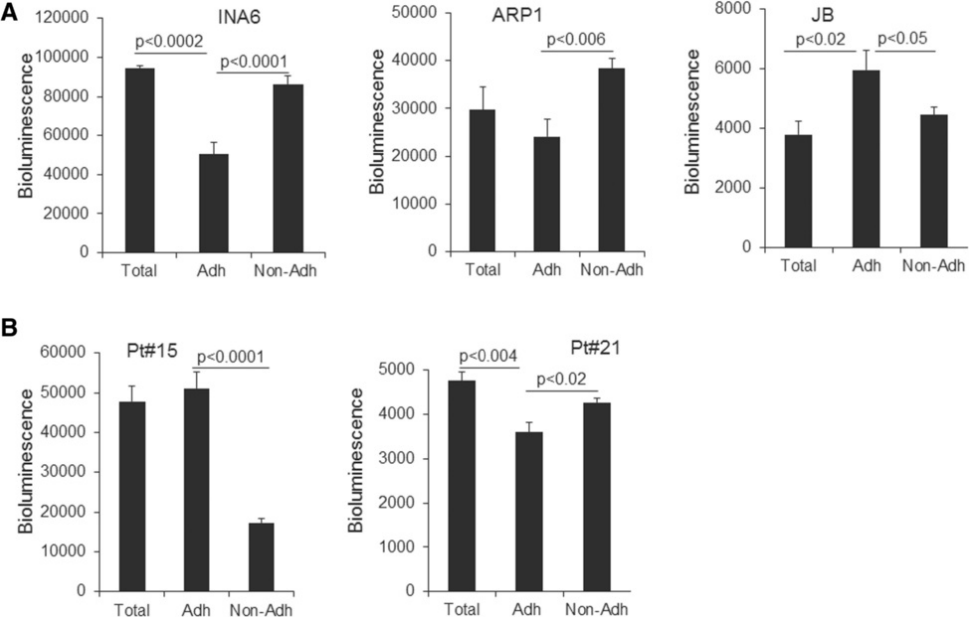
Adherent and nonadherent compartments of NBM coculture system support MM growth in a variable manner. Bioluminescence analysis of (**a**) luciferase-expressing MM lines (INA6, ARP1, JB) and (**b**) luciferase-expressing patient MM cells (maintained by passaging through SCID-rab mice; Pt#15, Pt#21) after 5 days of coculture; analysis of total NBM coculture (Total), adherent compartment only (Adh) or nonadherent compartment only (Non-Adh)

Because the NBM system contains adherent and nonadherent BM cells, we investigated whether these compartments support MM growth differently. After 7 days of NBM culture, we separated nonadherent BM cells from the adherent BM cells. MM cells expressing luciferase were cocultured with total NBM cultures or with the separate adherent and nonadherent portions; MM cell growth (based on bioluminescence) was compared after 5 days. Growth of IL-6-dependent MM line INA6 and stroma-independent line ARP1 was higher in coculture with nonadherent than with adherent NBM. In contrast, growth of the stroma-dependent line JB was higher in coculture with adherent than with nonadherent NBM (Fig. 3a). One of the patient’s MM cells were supported mainly by adherent NBM, but the other patient’s MM cells grew in all NBM compartments (Fig. 3b). These results suggest that the NBM system variably supports MM cell survival through its adherent and nonadherent compartments.

### Growth comparison of MM cells in coculture with NBM cells, osteoclasts and MSCs

**Fig. 4:**
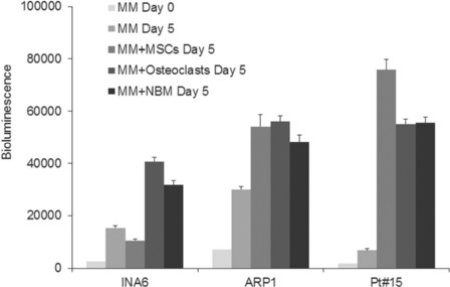
NBM system supports MM growth as efficiently as coculture models using MSCs or osteoclasts. Luciferase-expressing MM cell lines (INA6, ARP1) and patient MM cells (maintained by passaging through SCID-rab mice; Pt#15) were added to coculture models (MSCs only, osteoclasts only, NBM system); MM cells cultured alone (no BM cells) served as a control. Bioluminescence (indicative of MM cell number) was measured after 5 days; bioluminescence readings for MM cells on day 0 represent baseline

In vitro, BM stromal cells such as MSCs and osteoclasts have been shown to support MM cell survival and growth [7]. Therefore, we compared growth of MM cells in coculture with the NBM system with growth in other routinely used coculture models that contain single type of BM cells (MSCs or osteoclasts). Luciferase-expressing INA6 and ARP1 MM cell lines and luciferase-expressing primary MM cells each were cultured in equal numbers in the appropriate standard culture conditions and in coculture with MSCs, osteoclasts or the NBM system. All three coculture systems enhanced growth of MM cells compared to MM cells cultured alone (Fig. 4), and growth of all three was similarly sustained by coculture with osteoclasts and the NBM system. Coculture with MSCs variably supported growth of INA6 cells and patient MM cells compared to coculture with NBM cells and osteoclasts (Fig. 4). These results validate the NBM system as a reliable culture model that supports MM growth in vitro, similar to coculture models containing only osteoclasts or MSCs, and it simultaneously provides a platform for MM cells to interact with immune and stromal cells in BM.

### T cell-related cytokine secretion in the MM-NBM coculture system

**Fig. 5:**
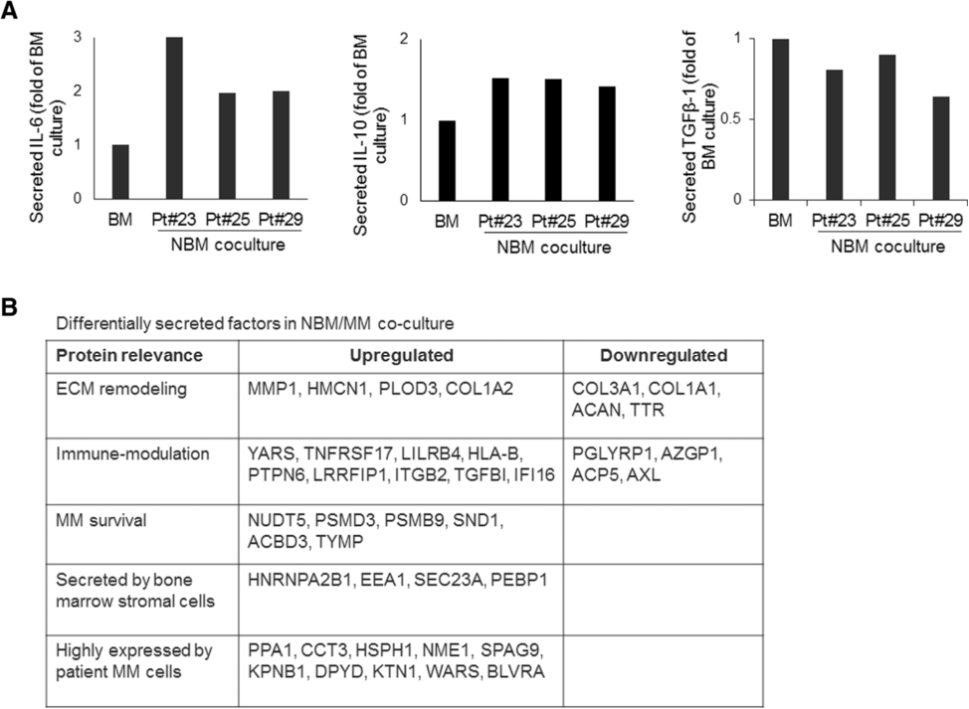
Coculture of MM cells in NBM system induces secretion of factors associated with modification of extracellular matrix and immune response. **a** Changes in cytokine (IL-6, IL-10, TGFβ-1) levels in conditioned media from 7-day coculture of three patients’ MM cells with NBM. Levels are shown as fold-increase of culture system without MM cells (BM). **b** Proteomics analyses identified factors in NBM/MM coculture supernatant (increased or decreased levels; *p* < 0.05; *n* = 3) compared to supernatant samples of BM cultured without MM cells or MM cells cultured alone. A thorough list is included in Additional file 4: Table S2

Cytokines play important role in MM cell growth and immune modulation in BM microenvironment, especially MM cell interaction with T cells [28]. We preformed cytokines array on conditioned media from NBM culture alone or coculture with patient MM cells after 7 days. Levels of IL-6 and IL-10 were elevated upon coculture of MM cells in NBM (Fig. 5a). TGFβ-1 secretion was slightly decreased upon coculture, while IL-2, IL-4, IL-5, IL-12, IL-13, IL-17A, IFN-γ, TNF-α and G-CSF were unchanged (data not shown). These data show sustained secretion of cytokines, previously reported in MM progression, during coculture of MM cells in the NBM system.

### Proteomics identified secreted factors in the NBM system

We examined proteins secreted by MM cells and BM microenvironmental cells within the NBM system. In order to determine the overall effect of MM and BM cells on each other within the NBM system, we performed protein profiling of the supernatant samples from the culture of MM cells or BM cells as well as the coculture of NBM/MM cells, using MM cells from 3 patients and BM from 3 different healthy donors. Cumulatively, 1843 different proteins were detected in at least one of the three groups. Of the 1843 proteins, 89 proteins were significantly changed in the NBM cocultures as compared to BM or MM cells cultured alone (Fig. 5b; Additional file 4: Table S2). These proteins included clinical MM markers B2M and LDHA, which were more abundant in the NBM/MM group than in NBM (>2–2.4 fold increase, *p* < 0.04). Seventy eight of the those 89 proteins were detected at high levels in the NBM coculture supernatants; these proteins were undetected or detected at low levels in the supernatants from BM or MM cells cultured alone. Similarly, 11 proteins were undetected or identified at low levels in the NBM coculture supernatants, in comparison to BM or MM cells cultured alone. These proteins differentially secreted in the NBM coculture system indicate changes in MM cells and BM microenvironmental cells that are induced by their effects on each other.

MM cells directly and indirectly impose changes in the BM milieu that can alter remodeling of the BM extracellular matrix and regulation of immune compartment [29]. MMP1, important for MM invasion and secreted by MM stromal cells, was present at increased levels in NBM coculture supernatants [30], as were HMCN1, a mediator of cytokinesis [31], and PLOD3, an extracellular matrix glycosyl transferase enzyme [32]. However, structural proteins of the extracellular matrix, such as COL3A1, COL1A1 and ACAN (a proteoglycan), were detected at lower levels in NBM coculture than in the BM cells cultured alone. Other factors upregulated in NBM coculture included proteins associated with alteration of antigen-presentation (LILRB4, HLA-B) [33], regulation of inflammation, and inhibition of T-cell response (ITGB2, LRRFIP1, PTPN6) [34–36]. Factors related to innate immune response (AZGP1, AXL) [37–39] were present at decreased levels in the NBM coculture supernatants. Increased levels of proteins associated with exosome miRNA loading (HNRNPA2B1) [40] and vesicular constitution (SEC23A, EEA1) [41, 42] indicate communication between stromal and MM compartments. Proteins important for tumor cell survival (SND1) [43], proteins known to be expressed by MM cells (PPA1, CCT3, NME1, SPAG9) [44–47], and a marker for bone resorption (DPYD) [48] were detected at higher levels in the NBM coculture supernatants. Together these findings indicate active communication among MM cells, immune cells and stromal cells in the NBM coculture system.

### Anti-MM efficacy of clinical agents in the NBM system

**Fig. 6:**
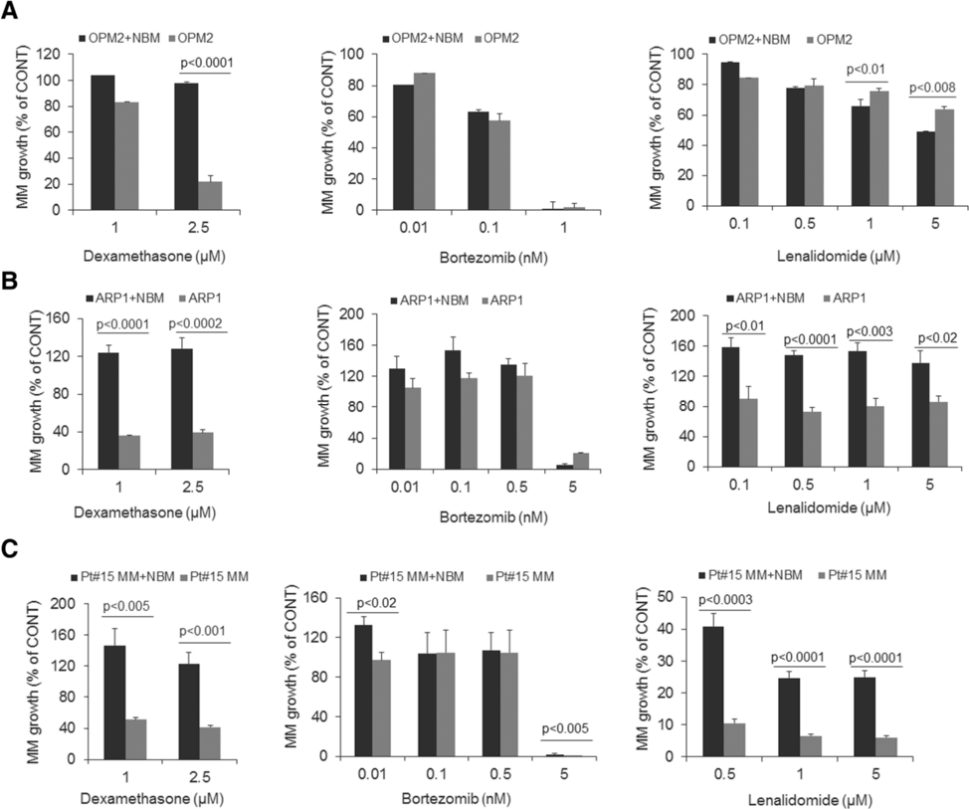
The NBM coculture system provides a platform to test MM cell response to clinical agents. Bioluminescence analysis of luciferase-expressing OPM2 (**a**), ARP1 (**b**) and patient MM cells (**c**; maintained by passaging through SCID-rab mice; Pt#15) cocultured in the NBM system (*black* bars) or cultured alone in their standard conditions (*gray* bars) and exposed to indicated concentrations of dexamethasone, bortezomib and lenalidomide. Data are represented as percent growth inhibition relative to the respective untreated MM cells (CONT; set to 100 %)

Because the BM microenvironment confers drug resistance to MM cells [29], we examined the effects of clinical anti-MM agents in the NBM system. We used the well-characterized OPM2 and ARP1 MM lines, which are capable of in vitro growth without cellular or cytokine support, as well as patients’ MM cells that have been maintained by passaging in a SCID-rab mouse; all types of MM cells used in these experiments express luciferase. MM cells were cultured alone or cocultured in the NBM system; cells were treated with dexamethasone (1–2.5 μM), bortezomib (0.01–5 nM) or lenalidomide (0.1–5 μM) for 5 days (Fig. 6). In the absence of these agents, overall growth of OPM2 and ARP1 cells was profoundly higher in the NBM system than in culture alone, based on bioluminescence analysis 5 days after initiation of cultures (see Fig. 2e). All three agents had variable effects on the growth of MM cells. Dexamethasone treatment inhibited growth of OPM2, ARP1 and the patient’s MM cells cultured alone but not in the NBM coculture system (Fig. 6a–c). Treatment with bortezomib resulted in growth inhibition of all MM cells in both culture settings at higher concentration (i.e., 5 nM; Fig. 6a–c). In the presence of lenalidomide, a lower proportion of OPM2 cells survived in the NBM system than in culture alone, which is consistent with the agent’s immunomodulatory properties (i.e., 1 and 5 μM; Fig. 6a). In contrast, lenalidomide treatment resulted in stimulation of ARP1 cell growth in the NBM coculture system and moderately inhibited growth when these cells cultured alone (Fig. 6b). Lenalidomide markedly inhibited growth of patient’s MM cells in both culture settings, although a higher proportion of residual MM cells were detected in the NBM coculture system (Fig. 6c). These data demonstrate that the NBM coculture system effectively supports MM cell survival and growth, but the anti-MM effects of clinical agents in this system are highly variable.

## Discussion

We demonstrated establishment of the novel NBM coculture system, where freshly obtained healthy donor BM cells are cultivated with serum from MM patients, followed by long-term (7 days) coculture with primary CD138-selected MM cells or stroma-dependent or -independent MM cell lines. Regardless of molecular features (e.g., risk signature or subset classification) and presence of allogeneic immune cells (e.g. cytotoxic T lymphocytes), MM cells from most cases survived and propagated in this system, and their growth was comparable to previously reported coculture systems. Interestingly, few MM cells survived in NBM containing healthy donor serum, use of patient serum increased MM cell survival and growth of tumor cells in the NBM coculture system, which contains typical BM cells mainly of hematopoietic lineages but also cells of mesenchymal lineages. Adherent and nonadherent compartments of the NBM system variably supported MM growth, and a variety of secreted immunomodulatory cytokines (e.g., IL-10) and growth factors (e.g., IL-6) were detected in the conditioned medium. This system is clinically relevant because it allows study of the immediate or initial response of adult whole BM to the invasion of MM cells and provides another platform for unraveling the mechanisms by which MM cells escape immune surveillance.

The NBM system offers advantages for studying clinically relevant aspects of patient MM cells and their microenvironment. It is different from an autologous BM setting that has already been affected by MM and has varying proportions of non-malignant cells in samples from different patients [10], and from coculture systems that are limited to a single type of microenvironmental cell [1–9]. It is also different from in vivo models for primary MM that use fetal bone, such as the SCID-hu model [11, 12], or that lack human hematopoiesis, such as the calcium phosphate-based scaffold [16]. The findings that growth of MM cells in the NBM system was comparable to their growth in coculture with the supportive osteoclasts or MSCs further underlines the advantage of this system for studying MM-microenvironment interactions due to use of a more comprehensive adult BM content, particularly immune cells.

The BM microenvironment protects MM cells from drug-induced cytotoxicity [29]. In this NBM coculture system, MM cells were protected from dexamethasone, which is similar to what is observed when MM cells are cocultured with MSCs or osteoclasts [24]. Bortezomib’s cytotoxic effects against MM cells were similar whether cells were cultured alone or cocultured in the NBM system. Immunomodulatory agent lenalidomide had contrasting effects on MM cell growth in the NBM coculture system, reflecting its ability to act on MM cells and immune cells. Lenalidomide had a greater anti-MM effect against OPM2 cells in the NBM coculture system than in culture of these cells alone. In contrast, lenalidomide stimulated growth of ARP1 cells in the NBM coculture system and moderately inhibited growth of these cells cultured alone, suggesting lenalidomide’s effects on the BM microenvironment variably impact growth of MM cells. Indeed, recent study demonstrated the paradoxical effect of lenalidomide on production of tumor-promoting cytokines and growth of MM cell lines [49]. Whereas lenalidomide has been shown to be beneficial for smoldering MM patients who are at risk for developing active disease and for MM patients with low-risk disease our findings emphasize the need to address the utility of IMiDs for MM patients with high-risk disease. Thus, the NBM coculture system reflects clinical MM by its ability to support survival of primary MM cells and variable anti-MM responses to clinical agents, along with its ability to evaluate simultaneous cytotoxicity on MM cells and BM cells.

The ability of primary MM cells from certain cases to cultivate and survive a within nonmyelomatous hematopoietic environment was previously shown in different systems [11–13, 50]. Healthy donor peripheral blood mononucleated cells preconditioned with IL-2 for 24 h were able to induce variable immune responses against different MM cell lines in a short bioluminescence-based assay [27]. We formally described this assay [23] and expanded its use for primary MM cells that are capable of passaging only in SCID-hu/SCID-rab models. In the syngeneic C57BL/KalwRij mouse model, MM cells (5TMM murine MM cell line or the more aggressive variant 5T33 cell line) are intravenously transplanted into syngeneic, immunocompetent, C57BL/KalwRij recipient mice and produce systemic MM [50]. It is likely that certain immune cells and factors were activated in response to MM in the 5TMM murine model [51] and in the NBM coculture system. Thus, the high variability in long-term survival of MM cells in this allogeneic system (Fig. 2b) could be attributed to both immune cell response and lack of a sufficiently supportive environment to induce the necessary survival signaling to cells of each case of primary MM. Alternatively, it is possible that, while a significant proportion of primary MM cells were initially targeted by immune cells in the NBM system, a resilient subpopulation of MM cells capable of survival (due to the presence of patient serum) cultivated the normal BM microenvironment to allow their propagation, analogous to dissemination of MM cells in new BM niches.

Although MM induces phenotypic changes to the cellular compartments of the BM and modifications to the extracellular matrix and cytokine milieu, there is a lack of in vitro studies to elucidate these localized molecular changes in the BM microenvironment. The NBM coculture model does not use artificial matrix coating (fibronectin, collagen and matrigel) to study matrix remodeling, but it shows that such factors can be secreted by the cocultured cells. The proteomic analysis of NBM supernatant, although descriptive, exposes known and some unknown molecules relevant to MM pathogenesis. In our system, we observed increased secretion of collgenase and MMP-1 and decreased secretion of collagens such as COL3A1 and COL1A1 [30]; this is consistent with conditions important for MM invasion in the BM niche. Matrix remodeling is further reflected by changes in high molecular weight extracellular proteins: increased HMCN1 [52] and decreased ACAN [53] in the NBM coculture conditioned medium. Increased detection of cell-surface receptor protein ITGB2 suggests dampening of the TLR-mediated myeloid cell inflammatory response [34], while increased LRRFIP1 indicates repressed monocyte production of pro-inflammatory cytokine, TNFα [35]. Upregulation of LILRB4, whose ligand is expressed by T cells, could be implicated in generation of tolerized dendritic cells through the action of cytokines IL-10 and interferon-α [33]. Although TNFRSF17, highly expressed by MM cells [54], is detected at increased level in NBM coculture supernatants and is considered a target for therapy involving CAR-expressing T cells. Increased levels of PTPN6 indicate negative regulation of T cell activity [36] in the NBM system. Downregulation of secreted factors such as PGLYRP1 [55], a pattern recognition protein, and AZGP1, which plays a role in MHC antigen processing [37], in the NBM coculture suggest a diminished innate immune response against invasive MM cells. While AXL is shown to inhibit TLR-mediated immune activity, it is also a soluble receptor for GAS6 [38], a survival factor expressed by MM cells [39]. We did not detect AXL in NBM coculture or MM cell monoculture, but it was abundant in BM cell monoculture, suggesting that the GAS-6/AXL axis may not promote proliferation of MM cells in an allogeneic BM setting. Communication between MM cells and mesenchymal cells was indicated by increased levels of HnRNPA2B1, a miR-15a loading protein found in exosomes derived from patient BM stroma [40, 56, 57]. Vesicular markers, such as EEA1 [42] and SEC23A [41], were upregulated in NBM cocultures, suggesting secretory activity in the BM stromal compartment. Some proteins detected in NBM cocultures may be associated with changes in surviving MM cells themselves (i.e., PPA1, CCT3, NME1, SPAG9); compared to normal plasma cells, we detected high gene expression of these factors in CD138-sorted cells from MM patients’ BM (data not shown) [44–47]. We also detected metabolism-related proteins (WARS, BLVRA) not previously reported to be secreted but upregulated in MM cells sensitive to anti-MM agents suggesting their role in MM survival in BM microenvironment [58, 59]. Altogether, we detected proteins related to immune deactivation, extracellular matrix modification and MM survival in the liquid milieu of the NBM coculture system, possibly providing clues about MM survival in an allogeneic BM microenvironment. Whether some of these abnormal changes persist at other clinical stages such as post-treatment and remission, and contribute to disease progression is a matter of continual study.

## Conclusions

We established the reproducible NBM coculture system in which healthy adult BM interacts with and supports growth and survival of primary CD138-selected MM cells. MM cells or subset of MM cells from most cases were capable of escaping immune surveillance and propagate in this system. Adherent and nonadherent compartments contributed to these effects, and patient serum was a necessary component. The NBM coculture system may be a useful model for studying immunologic and microenvironment aspects of newly colonized bone marrow and for testing clinically relevant anti-MM agents in vitro using patients’ MM cells.

### Consent statement

The Institutional Review Board of the University of Arkansas for Medical Sciences approved the research study. All subjects provided written informed consent in accordance with the Declaration of Helsinki. Signed Institutional Review Board–approved informed consent forms are on record and are available for review upon request.

## Additional files

Additional file 1: Figure S1. Flow cytometry based identification of MM cells in the NBM coculture system. MM cells in three representative NBM cocultures with primary MM cells identified by CD45/CD38-based flow cytometry analyses (left panel) or CD138 (right panel) phenotype on day 1 and 7 of coculture. Percent values represent gated MM cells. (TIF 172 kb) Additional file 2: Table S1. List of patient myeloma samples with detailed myeloma parameters. (XLSX 11 kb) Additional file 3: Figure S2. Long-term passaging of MM cells and patient serum as an essential component in the NBM. (A) Flow cytometry analyses showed prolonged survival of a primary MM cell sample (boxed population, CD45/CD38 MM cells) in the NBM coculture system. MM cells were passaged to freshly prepared NBM system every 2 weeks. (B) Growth of primary MM cells (samples from 2 different patients) in NBM coculture system established with serum from a healthy donor (left) or from a MM patient (right). MM cells were identified and quantified by CD45/CD38 flow cytometry analyses (boxed population indicates % MM). Numbers below the plots indicate the number of MM cells in total coculture population. (TIF 139 kb) Additional file 4: Table S2. Signal intensity of peptides detected by proteomics in NBM/MM coculture or MM or BM culture supernatants (*n* = 3). List ordered by highest to the lowest detection in coculture (NBM/MM) compared to MM or BM. (XLSX 21 kb)

## Abbreviations

MM: Myeloma
BM: Bone marrow
NBM: Normal bone marrow
MSCs: Mesenchymal stem cells
