# Supplementary figures and images for: Primary myeloma interaction and growth in coculture with healthy donor hematopoietic bone marrow
Source: BMC Cancer. 2015 Nov 6;15:864. doi: 10.1186/s12885-015-1892-7 (PMC4636897; doi:10.1186/s12885-015-1892-7)

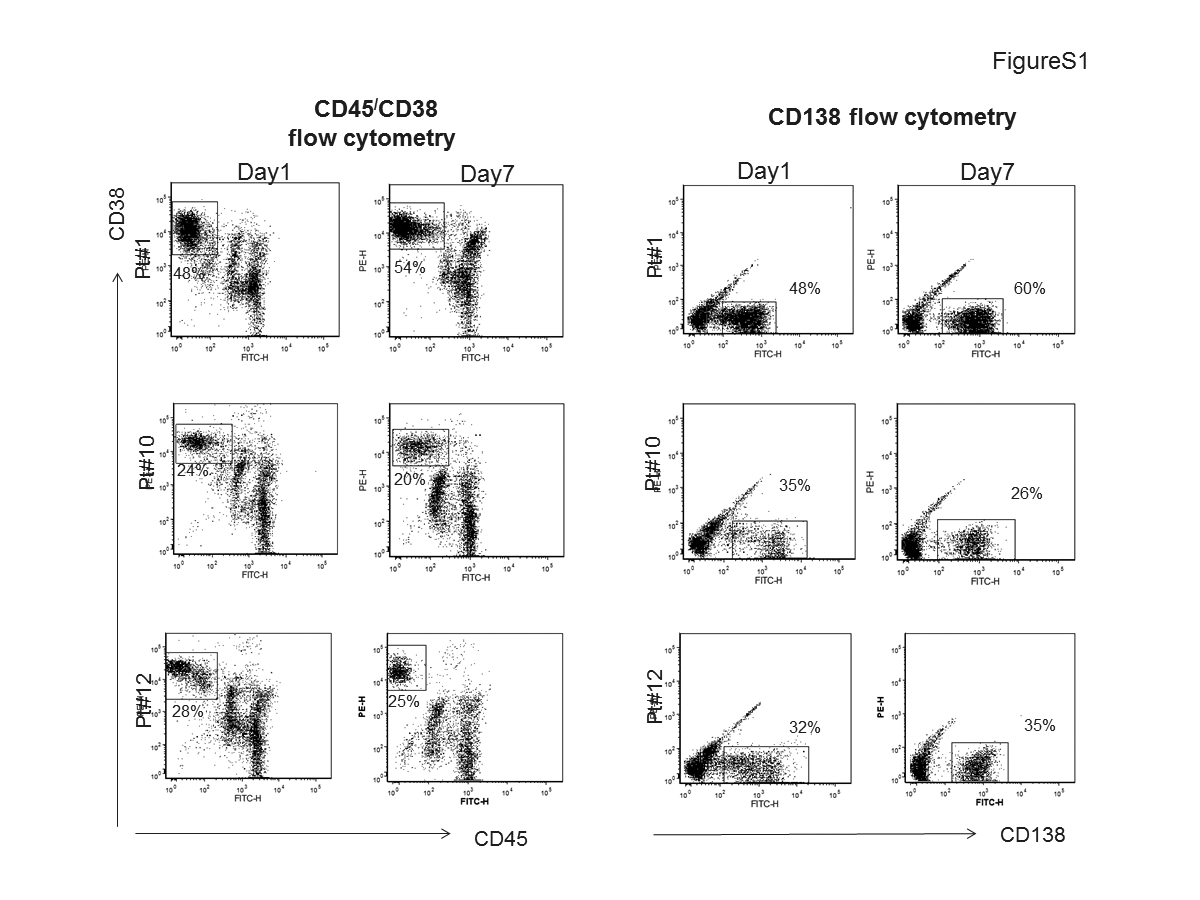

Supplement: Additional file 1: Figure S1. — Flow cytometry based identification of MM cells in the NBM coculture system. MM cells in three representative NBM cocultures with primary MM cells identified by CD45/CD38-based flow cytometry analyses (left panel) or CD138 (right panel) phenotype on day 1 and 7 of coculture. Percent values represent gated MM cells. (TIF 172 kb) [file 12885_2015_1892_MOESM1_ESM.tif]

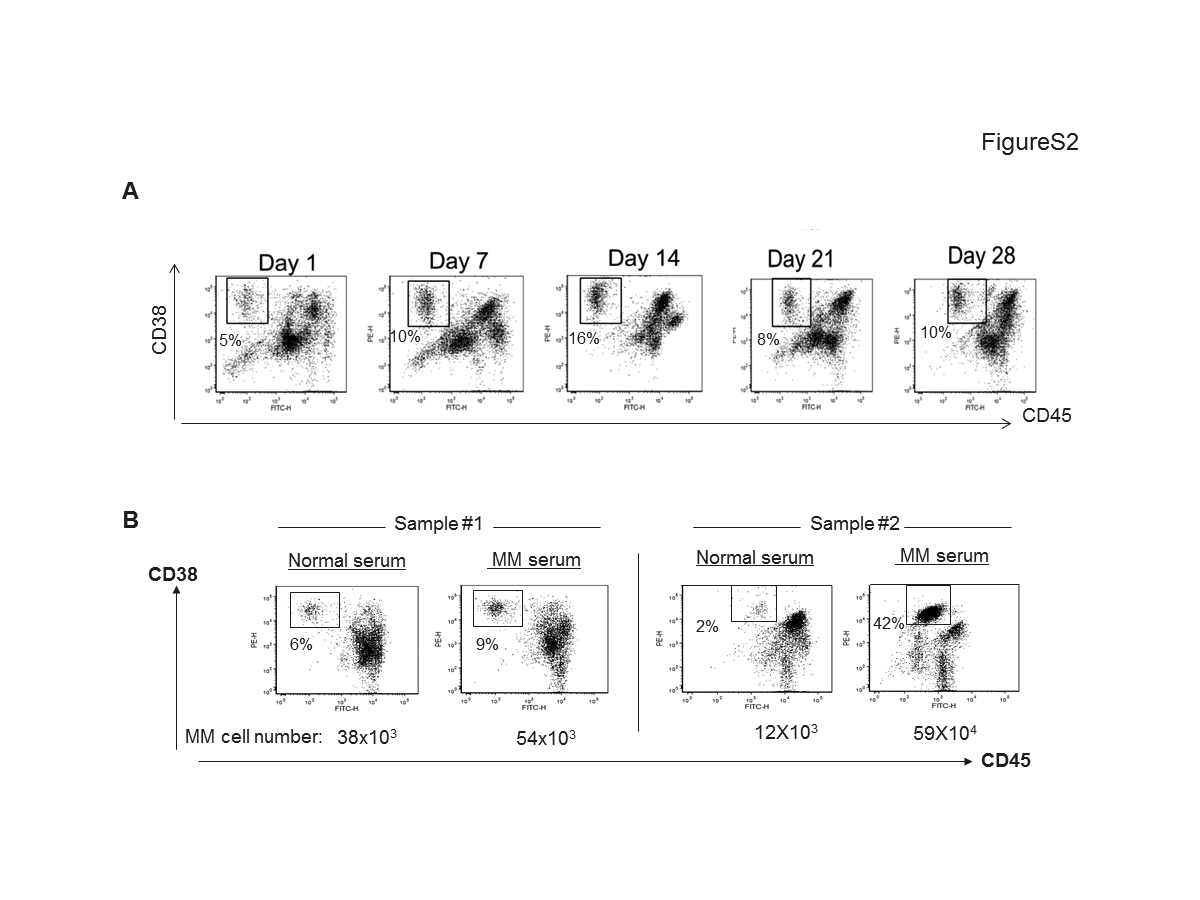

Supplement: Additional file 3: Figure S2. — Long-term passaging of MM cells and patient serum as an essential component in the NBM. (A) Flow cytometry analyses showed prolonged survival of a primary MM cell sample (boxed population, CD45/CD38 MM cells) in the NBM coculture system. MM cells were passaged to freshly prepared NBM system every 2 weeks. (B) Growth of primary MM cells (samples from 2 different patients) in NBM coculture system established with serum from a healthy donor (left) or from a MM patient (right). MM cells were identified and quantified by CD45/CD38 flow cytometry analyses (boxed population indicates % MM). Numbers below the plots indicate the number of MM cells in total coculture population. (TIF 139 kb) [file 12885_2015_1892_MOESM3_ESM.tif]
